# Design and Implement Strategy of Wireless Bite Force Device

**DOI:** 10.3390/bioengineering10050507

**Published:** 2023-04-23

**Authors:** Jinxia Gao, Zhiwen Su, Longjun Liu

**Affiliations:** 1 Key Laboratory of Shaanxi Province for Craniofacial Precision Medicine Research, College of Stomatology, Xi’an Jiaotong University, Xi’an 710004, China; gaojinxia@xjtu.edu.cn; 2 Department of Prothodontics, College of Stomatology, Xi’an Jiaotong University, Xi’an 710004, China; 3 Institute of Artificial Intelligence and Robotics, The School of Electronic and Information Engineering, Xi’an Jiaotong University, Xi’an 710049, China

**Keywords:** stress, occlusal splints, printing, machine learning, engineering, osteoarthritis

## Abstract

Abnormal bite force is an important risk factor for oral and maxillofacial disorders, which is a critical dilemma that dentists face every day without effective solutions. Therefore, it is of great clinical significance to develop a wireless bite force measurement device and explore quantitative measurement methods to help find effective strategies for improving occlusal diseases. This study designed the open window carrier of a bite force detection device through 3D printing technology, and then the stress sensors were integrated and embedded into a hollow structure. The sensor system mainly consisted of a pressure signal acquisition module, a main control module, and a server terminal. A machine learning algorithm will be leveraged for bite force data processing and parameter configuration in the future. This study implemented a sensor prototype system from scratch to fully evaluate each component of the intelligent device. The experimental results showed reasonable parameter metrics for the device carrier and demonstrated the feasibility of the proposed scheme for bite force measurement. An intelligent and wireless bite force device with a stress sensor system is a promising approach to occlusal disease diagnosis and treatment.

## 1. Introduction

The masticatory system consists of the masticatory muscles, temporomandibular joints, and teeth [1]. Bite force is the synergistic and interactive force generated by the upper and lower teeth, joints, and muscles in the masticatory system, and is an important indicator determining the functional state of the masticatory system [2]. When the upper and lower teeth cannot form a dynamic balance with the entire masticatory system, abnormal activities can occur, resulting in abnormal forces and eventually leading to occlusal-related diseases [3].

Among the various occlusal-related diseases, taking temporomandibular joint osteoarthritis (TMJ-OA) as an example, a number of studies have shown that abnormal bite force is a critical factor inducing TMJ-OA [4]. TMJ-OA occurs in the orofacial region and is characterized by joint pain, swelling, and stiffness, etc. [5,6,7]). To date, TMJ-OA is still the most common destructive oral and maxillofacial disease affecting quality of life and is also one of the most difficult problems faced by dental practitioners [8]. Previous studies have confirmed that mechanical stress overloading is a key factor in inducing osteoarthritis lesions [4,5,9]. Physiological loading conditions can promote the adaptive remodeling of joints, and abnormal remodeling and even the formation of osteoarthritis can eventually occur when the temporomandibular joint is overloaded for a long time or its adaptability is reduced.

Molecular signaling and pathological studies of temporomandibular joint osteoarthritis have shown that it can directly lead to mechanical damage to the joint tissue when the temporomandibular joint is subjected to abnormal or excessive stress, such as physical damage to cells. Moreover, the temporomandibular joint exhibits a hypoxic state when sustained and excessive mechanical load is applied. Vascular endothelial growth factor (VEGF) is activated following the activation of hypoxia-induced factor-1 (HIF-1), which further regulates the disordered expression of matrix metalloproteinase (MMP-13) and tissue matrix metalloproteinase (TIMP-1), resulting in an imbalance in the synthesis and distribution of extracellular matrix, eventually leading to the destruction of articular cartilage [10,11]. In addition, reoxygenation is observed when the mechanical loading on the joint is reduced. Joints release free radicals during hypoxia and reperfusion cycles, which leads to joint degeneration and damage [10,12,13]. In summary, according to the mechanotransduction process, the mechanical force applied to the mandibular condyle cartilage is finally converted into molecular signals in cells and triggers morphological and phenotypic changes. Therefore, it is crucial to study the quantitative detection method of different levels of bite force to further reveal the occurrence of temporomandibular joint osteoarthritis.

Measuring the bite force in occlusal conditions is not a simple task and has a history of more than 100 years. With the development of emerging technologies in engineering, the size of electronic measurement devices has greatly reduced and made it possible to implant strain gauges into complete dentures and restorations. Devices that detect bite force from the perspective of electronic sensors such as resistance, voltage, and capacitance have been extensively reported [14,15]. Existing studies have mainly focused on the measurement of bite force, but there is still a lack of in-depth research on how to rationally use the large amount of detected occlusal force data to guide clinical and scientific practice. In recent years, the application of artificial intelligence in dentistry has also been widely discussed, and transdisciplinary research may become more important [16,17]. In the future, benefiting from the development of artificial intelligence technology, patients may be able to achieve self-monitoring and management of oral-related diseases by wearing a portable intelligent device that can collect continuous oral bite force and perform deep learning and neural network analysis. For this reason, designing an artificial intelligence algorithm model based on a large amount of experimental data, monitoring, and feedback through calibration is useful for assisting clinical disease diagnosis and treatment.

Preliminary studies have found that occlusal stabilization splints (OSS) are an important method for the treatment of temporomandibular joint disorders [18]. If a piezoelectric film sensor and control chip are embedded in the OSS effectively, bite force data can be collected in real time, transmitted, and stored to the server of the mobile phone, which can monitor the bite force of the experimental object continuously. Research on the intelligent detection of quantitative bite force devices has important auxiliary significance for revealing the intrinsic relationship between the quantitative bite force and changes in TMJ osteoarthritis, whether the relationship shows regularity, and what kind of regularity exists. To address these problems, the aim of this study is to explore a wireless detective and intelligent analysis device for bite force. In this experiment, the chips used for force measurement in the engineering field will be embedded into the modified stabilization occlusal splint that fabricated through 3D printing technique in dental filed. The quantitative and wireless transmission of bite force will be completed by using a pressure-sensing chip, Bluetooth transmission, and other technologies. We expect that the finished detector has an important guiding role for quantitative detection, the intervention of bite force, and the diagnosis and treatment of occlusal diseases.

## 2. Materials and Methods

### 2.1. Designing and Manufacturing of the Bite Force Detector

**Figure 1:**
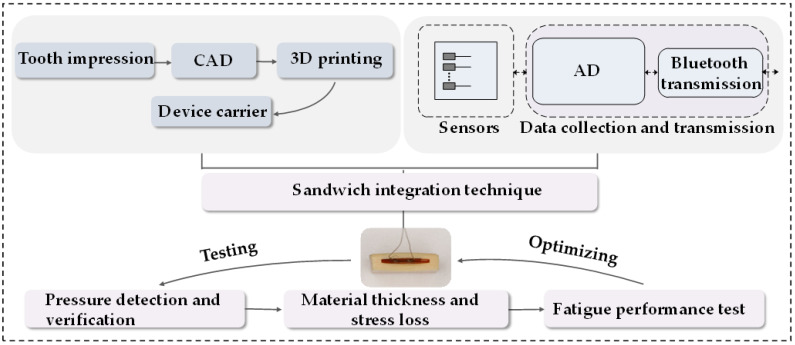
Designing and manufacturing schematic of the bite force detector by sandwich integration technique.

**Figure 2:**
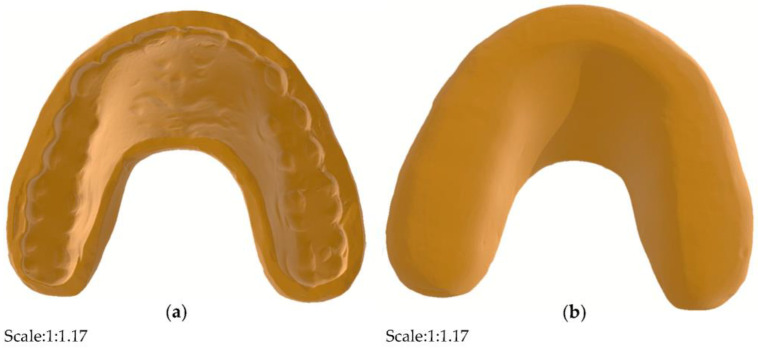
Modified occlusal splint. (**a**) scanning images in tissue surface view; (**b**) scanning images in occlusal view.

**Figure 3:**
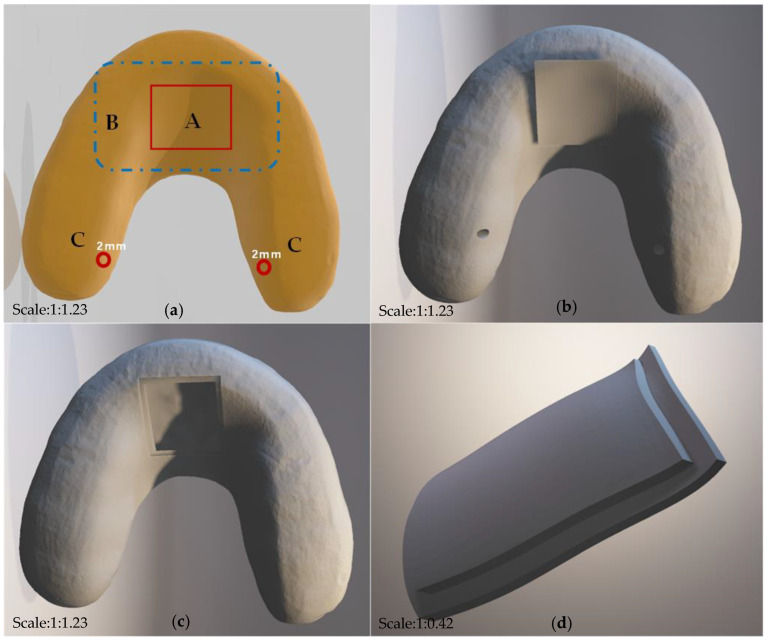
Hollow structure design for the detective device. (**a**) schematic diagram of the device: A was designed as a detachable opening window; B was an extension of area A; C was the occlusal surface region; (**b**) 3D design of the device; (**c**) open window in the palatal section; (**d**) accessory for the open window structure.

In this study, the design and testing schematic of the detector are shown in the figure below (see in Figure 1). A pair of human standard maxillary and mandibular dentition models that are commonly used in the dental laboratory were selected for detector design. In this experiment, the original main structure of the detector on the maxillary dentition model was designed by using a light-cured resin material (HUGE, Shandong Huge, Shanghai, China) for individual trays. The 3D images of the modified splint were scanned by using an intraoral scanning system (DS-EX Pro, Shining3D, Hangzhou, China), and the data were saved in the STL. format and designed by using computer-aided design (CAD) software (see in Figure 2). The device was designed with a hollow structure for the insertion of accessories and fully covered the entire dental arch and the palatal surface, which was actually an improved version of the occlusal stabilization splint (see in Figure 3). In addition, the hollow splint needed to form a penetration structure on the lingual surfaces of the bilateral maxillary posterior teeth to facilitate the embedding of the chip material in the later stages. The distance between the occlusal surface of the splint and the opposite teeth should be set to zero to achieve ideal occlusal contact under different conditions [19]. In this experiment, the upper and lower layers of the main structure of the detector were set to a thickness of approximately 1.5 mm. The data from the CAD technology were imported into a 3D printer (SLA600, ZRapid Technologies Co., Ltd., Suzhou, China) and then fabricated with resin composite materials (material 9400, Dongguan Aide Polymer Material Technology Co., Ltd., Guangzhou, Guangdong, China) using additive manufacturing techniques. This idea was first proposed in 2019 [19], and the exploration of local bite force was carried out in 2021 [20]. This study was designed on the basis of previous research, while the biggest difference from the previous study was that this study mainly focused on full dentition bite force detection.

### 2.2. Sensor Assembly and Testing

**Figure 4:**
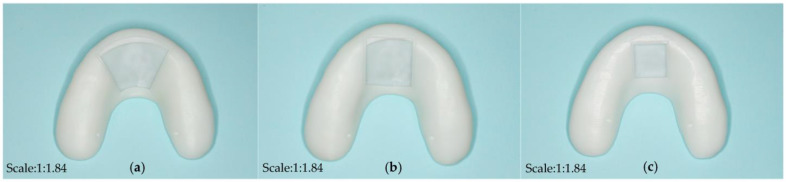
Different design of open window structure. (**a**) flabelliform window; (**b**) rectangle window (length × width: 25 mm × 15 mm); (**c**) rectangle window (length × width: 15 mm × 15 mm).

**Figure 5:**
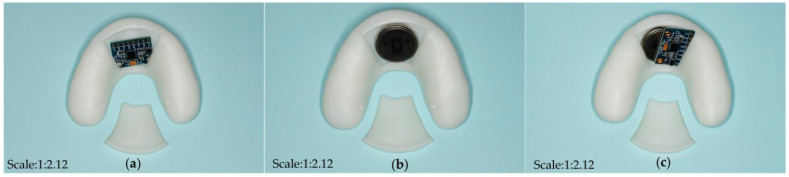
Assembly and embedding. (**a**) embedment of pressure chip and control chip; (**b**) button battery could be effectively embedded into the flabelliform window structure; (**c**) chip and battery could not be embedded in the flabelliform window section completely.

**Figure 6:**
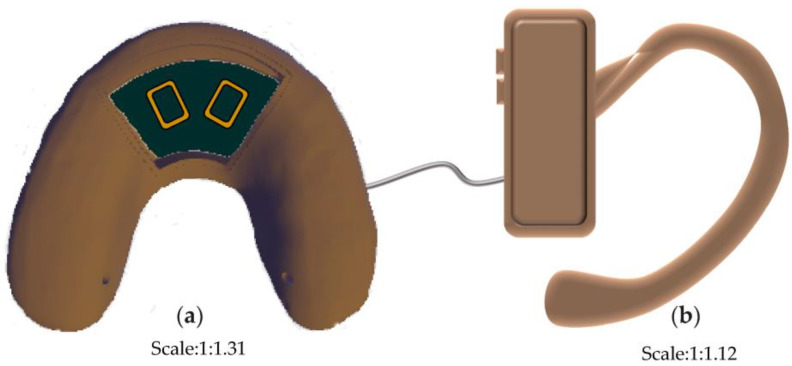
Modified portable bite force detection device. (**a**) chip is embedded in the hollow structure; (**b**) battery and controller can be embedded in external structure hanging on the ear.

**Figure 7:**
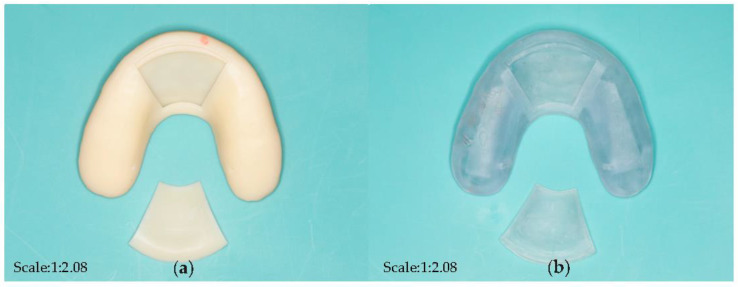
Carrier of the detector with different dental materials. (**a**) ultraprint dental temp CB UV; (**b**) ultraprint dental soft splint UV with transparency and inferior hardness features.

Different open window structures were designed to fit the various sizes of commercially available chips and batteries (Figure 4). In this experiment, first, components such as sensitive piezoresistive chips (HuaLanHai, BHF350-3AA, Guangzhou, China), an ultralow-power MCU (Nordic Semiconductor, NRF52832, Oslo, Norway), a multichannel analogue-to-digital converter (ADC) (Nordic Semiconductor, SAADC, Oslo, Norway), and a button battery (Panasonic Semiconductor, Panasonic CR2032, Celebes, Indonesia) were assembled and embedded in the hollow structure of the detector (Figure 5 and Figure 6). Specially, the force sensor was composed of polycool film, a highly conductive material with excellent comprehensive mechanical properties, and a nanoscale pressure-sensing material. The bottom layer was a flexible film and a conductive layer on the composite, and the top layer was a flexible film and a pressure-sensing material on the composite. The two were attached by double-sided glue, and the sensing area of the upper and lower layers were isolated. When the induction zone was pressurized in the bottom layer of each separated conduction line, the output resistance of the metal port under different pressures changed accordingly. The force sensor could output corresponding voltage changes with dental pressure variations. It was easy to integrate into the bite force sensor structure with our strategy. Second, the accessory for the open window structure could be carefully encapsulated to the hollow position of the detector by laser welding technology to ensure that the detector could function effectively in an oral environment. Third, the information acquisition module was designed as follows: the voltage signal was captured and then converted to a pressure signal by a customized signal acquisition controller; meanwhile, the stress information was sent to the server module through Bluetooth 4.0 to realize the force detection, collection, data conversion, and Bluetooth transmission. Continued testing and optimization will be conducted in the selection of various materials. Carrier of the detector with different dental materials were selected and tested (Figure 7).

### 2.3. Verification and Calibration of the Pressure Detection Device

**Figure 8:**
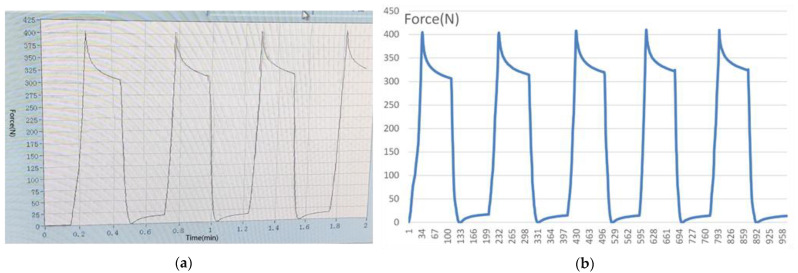
Pressure loading test. (**a**) parameter setting for electronic universal testing; (**b**) pressure data variation stored through customized signal acquisition controller.

In this section, the pressure detector was tested and verified by an electronic universal testing machine, the detailed technique of which was described in a previously published article in 2021 [20] (Figure 8). The fabricated detector was worn on the maxillary dental model, and the mechanical pressure loading test could be carried out according to the computer-aided program and equipment.

Meanwhile, the mechanical stress signals could be collected and stored in the mobile application. In the mechanical loading test, the range of the loading force was set to 0–500 N, and 400 N was set as the high warning value to simulate the abnormal bite force in oral conditions. When the loaded force was larger than or equal to the threshold of 400 N, the app generated an alarm while recording.

## 3. Results

### 3.1. The Main Structure of the Detector Based on the Sandwich Principle

In this section, the light-cured resin material commonly used in the clinic for individual trays was selected for the design of the improved occlusal stabilization splint on the standard maxillary dentition model. The full dental arch and the anterior part of the palatal surface were covered with a resin material, and the coverage on the buccal and labial sides of the maxillary dentition was approximately 2 mm, providing basic retention. In addition, the improved splint extended in the anterior of the maxillary palatal surface, showing a horseshoe shape, which was mainly designed for accessories containing batteries and control chips. Figure 2 illustrates the modified occlusal splint initially manufactured.

The main structures of the detector were marked as A, B, and C (Figure 3). Area A was designed as a detachable opening window, which mainly facilitated the embedding of materials. Area B was an extension of area A, which was convenient for accommodating batteries, control chips, etc. The thickness of the maxillary palatal hollow structure (area A and area B) was set to 3 mm, which was connected with the dentition hollow structure. The thickness of the hollow structure on the occlusal surface region (area C) should be 0.8 mm, and the width was designed to be more than 6 mm. Circular holes with a diameter of 2 mm were designed on the bilateral posterior molar area and connected to the hollow structure, which was convenient for fixing the film to the expected position. In this experiment, light curing resin was used to seal all the opening edges.

### 3.2. Chip Assembly, Data Collection, and Wireless Transmission

Three different kinds of open window structures were designed in this section (Figure 4). Figure 4a shows the occlusal splints with flabelliform windows. Figure 4b shows the occlusal splints with a large rectangular window (length × width: 25 mm × 15 mm). Figure 4c shows the occlusal splints with small rectangular windows (length × width: 15 mm × 15 mm). Occlusal splint integration with a circuit board and battery was not completely successful due to the size of the control chip and battery (as shown in Figure 5). Therefore, we improved the design by adding an extra oral structure hanging on the ear, on which the battery and controller could be embedded into the external device (as shown in Figure 6). As a continuous improvement experiment, we also selected another two different materials to print the carrier of the detector through 3D techniques (Figure 7a,b). The materials were used for occlusal splint fabrication in dental clinical practice. In addition, the biosensor showed effective performance for pressure detection at local sites during a mechanical pressure loading test in a previous experiment, as mentioned in the article [20] (Figure 8). The pressure data were collected and transmitted by the Bluetooth protocol. The app could load the pressure data and visualize the data on smartphones and computers. Occlusal pressure collection, wireless transmission, and data analysis methods were similar to the approach described in our previous article [19,20].

## 4. Discussion

This experiment designed a hollow carrier bite force detective device by using 3D printing technology, which is a further extension and improvement of the previous conception and exploration of the research group in the intelligent analysis of wireless bite force device research. The detector is not yet a very complete and precise force measurement device, while the ideas and fusion technologies formed by this study have immense potential for the diagnosis and treatment of oral and maxillofacial diseases, including temporomandibular joint osteoarthritis [21]. Although the existing research is interesting and full of potential, the following problems still need to be overcome in follow-up research. The discussion of the design features of the bite force detector: Researching the quantitative measurement device of force continuously is significant for further the preventing and controlling of force-related dental diseases [22]. As mentioned before, the main body of the bite force detector was designed as a modified occlusal stabilization splint in this trial, which was based on the following considerations: first, the designed detector could be set on the patient’s maxillary dentition and obtained a certain retention, which was convenient for detecting the real-time force in occlusal conditions; on the other hand, this wearable oral delivery device would not only realize the detection of bite force but also had a therapeutic function since the occlusal stabilization splint also had the effects of improving temporomandibular joints and other occlusion-related diseases; third, after a slight improvement of the occlusal stabilization splint, it could cover the entire dentition and the maxillary palatal surface; meanwhile, the hollow structure in the main body should be designed to provide sufficient space for the insertion of batteries, sensors, controllers, etc. The pressure chip was mainly placed on the occlusal surface of the bilateral dentition to facilitate the collection of bite force under occlusal conditions. The button battery and control chip were mainly embedded in the anterior part of the maxillary palatal surface, making effective use of the maxillary cavity structure and, simultaneously, minimizing the foreign body sensation and discomfort of the patient. The above considerations and designs were well integrated with existing 3D printing techniques, chips, wireless transmission technologies, and intelligent algorithms into the oral field, providing ideas for the diagnosis and treatment of oral diseases.

The discussion of the material selection for 3D printing: As mentioned above, the main body of the detector was designed with a hollow structure, which was mainly used for the embedding of accessories such as pressure-sensing chips and batteries, which entailed higher requirements for the selection of 3D printing resin materials. The material for this experiment was industrial resin initially, which could not be used in patients directly. Moreover, residual monomers after polymer resin material curing may lead to certain side effects on oral health [23]. Therefore, for 3D printing carriers, it was necessary to find someone with better biological safety and stronger mechanical properties to replace the existing materials further. Until now, we have cooperated with the Modern Dental Group to print two different detectors (UltraPrint Dental Temp C&B UV, Guangzhou, China; UltraPrint Dental Soft Splint UV, Gibbstown, NJ, USA) through 3D printing techniques. Similarly, the carrier of the detectors was fabricated by selecting the dental material used for the stabilization of the occlusal splint since the carrier of the detector was modified from the occlusal splint. Therefore, the selection of carrier materials for detectors has made great progress in terms of biocompatibility. According to previous research, the occlusal splint should be worn for 6 months and only at night for treatment [24]. The modified occlusal splint (device) had both bite force detection and therapeutic effects; thus, the recommended wearing time of the detector was the same as that of the occlusal splint. Previous studies had shown the maximum bite forces in the molar regions in healthy adults were between 300 and 600 Newtons (N) [25]. In the mechanical loading test in our previous experiment, the range of the loading force was set to 0–500 N, and 400 N was set as the high warning value to simulate the abnormal bite force in oral conditions. It is very important to evaluate the flexural resistance and sensitivity of the device under different material and thicknesses in the future. 

The discussion of the accessory selection: The general principle of the design and selection of the internal accessories was to minimize the size of the detector while ensuring the normal function of the detector. The size of the detector was controlled to avoid evident occlusal interference and discomfort to the patient. The length, width, and thickness of the stress-sensitive chips selected in this experiment should be smaller than 8 mm, 6 mm, and 0.3 mm, respectively, which could be effectively embedded in the hollow structure of the occlusal surface of the bilateral posterior teeth of the detector. Research has shown that printing the sensor by using 3D electronic printing methods such as aerosol jet printing will be helpful for developing more personalized sensors [26,27,28]. Moreover, the micro battery selected for this experiment should be 0% Hg silver oxide with a rated voltage of 3.0 V and a size of 20 mm × 2 mm (diameter × thickness). The button-shaped battery could be embedded into the hollow section of the palatal surface of the detector by a special design. The lack of space was still a limitation when the controller and battery were embedded together. Therefore, size was a critical consideration in all accessory selection processes except for biosafety. With the development of the battery industry, an increasing number of smaller portable batteries could be integrated into our sensor system.

The discussion of the intraoral delivery and occlusal adjustment: The thickness of the occlusal surface of the improved bite force detector in this study remained even and ideal since the experiment was mainly completed in vitro. However, the ultimate purpose of this bite force detector was to be placed in the mouth for real-time bite force detection in future research. Considering that any abnormal occlusal interference by jaw movement will have adverse consequences on the patient’s oral maxillofacial system [29], the occlusal splint must be finely adjusted after delivery to ensure that the adjusted occlusal splint could not only detect the bite force but also have a positive improvement effect on the temporomandibular joints and other tissues of the research subjects [30]. Therefore, comprehensive and careful occlusal adjustment after wearing the modified occlusal plate was very important, and it was also an important approach to avoid iatrogenic damage to the temporomandibular joint.

The discussion of the artificial intelligence algorithm designing and data training: A pressure test could be performed by setting the designed bite force detector on the extraoral model, loading the computer program and applying different degrees of pressure through the universal testing machine. The detector could collect stress data synchronously and send the data to a computer. Mechanical stress data of the research object within a fixed period of time were analyzed by computer, and, finally, a biological force diagram was drawn. Artificial intelligence algorithm models could make predictions about oral diseases [31], thus it was speculated that an artificial intelligence algorithm model designed based on a large amount of experimental data could predict the condition of temporomandibular joint osteoarthritis in the future [32]. In addition, the existing detection devices were mainly focused on the measurement of bite force, while how to make reasonable use of the huge, detected bite force data and guide clinical and scientific research practice is full of potential for the dentistry field and needs to be further explored.

## 5. Conclusions

This study designed the open window carrier of a bite force detection device through 3D printing technology, and then the stress sensors were integrated and embedded into the hollow structure. This experiment provided technical support for the detection and intelligent analysis of wireless bite force device research. The experimental results showed reasonable parameter metrics for the device carrier and demonstrated the feasibility of the proposed scheme for bite force measurements. The intelligent and wireless bite force device with a stress sensor system was a promising approach to occlusal disease diagnosis and treatment. Authors should discuss the results and how they could be interpreted from the perspective of previous studies and of the working hypotheses. The findings and their implications should be discussed in the broadest context possible. Future research directions may also be highlighted. For intelligent algorithms, five important characteristic parameters related to occlusion per unit time will be extracted, including the average bite force, time, contact area, number of contact points, and animal pain level. Then, a one-dimensional deep convolutional neural network was used for intelligent analysis in the algorithm design of this project. The residual structure of the neural network was used to improve the test accuracy of the network. Before the neural network algorithm was trained, the researcher needed to calibrate the input data and the degree and level of the corresponding occlusal adjustment so that the algorithm model first obtained the network parameters on the calibrated dataset and then conducted the actual data based on the learned model parameters test.

## Data Availability

The data presented in this study are available on request from the corresponding author. The data are not publicly available due to privacy restrictions.
